# Zinc stress induces copper depletion in *Acinetobacter baumannii*

**DOI:** 10.1186/s12866-017-0965-y

**Published:** 2017-03-11

**Authors:** Karl A. Hassan, Victoria G. Pederick, Liam D. H. Elbourne, Ian T. Paulsen, James C. Paton, Christopher A. McDevitt, Bart A. Eijkelkamp

**Affiliations:** 1 Department of Chemistry and Biomolecular Sciences, Macquarie University, Sydney, NSW Australia; 2 Research Centre for Infectious Diseases, School of Biological Sciences, University of Adelaide, Adelaide, SA Australia

**Keywords:** Acinetobacter, Zinc, Copper, Transporter, Oxidative stress, Fatty acids, Macrophages, Membrane

## Abstract

**Background:**

The first row transition metal ions zinc and copper are essential to the survival of many organisms, although in excess these ions are associated with significant toxicity. Here, we examined the impact of zinc and copper stress on *Acinetobacter baumannii*, a common opportunistic pathogen.

**Results:**

We show that extracellular zinc stress induces a copper-specific depletion phenotype in *A. baumannii* ATCC 17978. Supplementation with copper not only fails to rescue this phenotype, but further exacerbates the copper depletion. Extensive analysis of the *A. baumannii* ATCC 17978 genome identified 13 putative zinc/copper resistance efflux pumps. Transcriptional analyses show that four of these transporters are responsive to zinc stress, five to copper stress and seven to the combination of zinc and copper stress, thereby revealing a likely foundation for the zinc-induced copper starvation in *A. baumannii*. In addition, we show that zinc and copper play crucial roles in management of oxidative stress and the membrane composition of *A. baumannii*. Further, we reveal that zinc and copper play distinct roles in macrophage-mediated killing of this pathogen.

**Conclusions:**

Collectively, this study supports the targeting of metal ion homeostatic mechanisms as an effective antimicrobial strategy against multi-drug resistant bacterial pathogens.

**Electronic supplementary material:**

The online version of this article (doi:10.1186/s12866-017-0965-y) contains supplementary material, which is available to authorized users.

## Background

*Acinetobacter baumannii* is a Gram-negative opportunistic human bacterial pathogen that is ubiquitous in hospital environments [1, 2]. Although genetically distinct from their nosocomial counterparts, community-acquired *A. baumannii* isolates are increasingly being recognized as serious threats to human health [3–5]. The *A. baumannii* pan-genome features a broad arsenal of antibiotic resistance determinants [6] and its genomic plasticity has also led to major differences in genes that play a role in persistence and virulence between strains [2, 4, 7–11]. Thus, the success of this human pathogen can, at least in part, be attributed to the ability of *A. baumannii* to readily incorporate foreign genetic material.

Although the mechanisms involved in the acquisition of essential transition metals ions, such as zinc (Zn) and iron (Fe), have been studied in detail in *A. baumannii* [12–19], how this organism responds to extracellular metal ion stress and intoxication remains poorly understood. In *A. baumannii* the first row transition metal ions Zn and copper (Cu) have critical roles in numerous cellular processes and are essential for viability [12, 13, 20]. However, the ability of Zn and Cu to form highly stable complexes with proteins necessitates their cellular abundance be tightly regulated to prevent intoxication that would lead to the inappropriate and highly detrimental binding of these ions to non-cognate metal binding sites, such as the Fe-S clusters of metalloproteins [21–23]. The molecular basis of Zn toxicity is multifactorial with Zn intoxication leading to perturbed transition metal ion homeostasis, impairment of oxidative stress response mechanisms [24, 25] and disruption of central carbon metabolism [26]. In the Gram-positive human pathogen *Streptococcus pneumoniae* extracellular Zn has been shown to compete for binding to the manganese (Mn)-recruiting lipoprotein PsaA, resulting in inhibition of Mn acquisition [25, 27]. As Mn is the primary co-factor for the sole known superoxide dismutase in *S. pneumoniae*, Zn-stressed, and consequently Mn-starved, *S. pneumoniae* cells are hyper-susceptible to oxidative stress [27, 28]. By contrast with Zn, Cu toxicity has typically been associated with its redox activity and potential to generate reactive oxygen species. However, in isolation Cu intoxication has been shown to be insufficient to induce oxidative stress [22]. Despite this, at the host-pathogen interface, Cu in combination with other factors, such as hydrogen peroxide, can contribute to antimicrobial oxidative stress [29–31]. The human innate immune system has also been shown to exploit the antimicrobial activity of metal ions with recent studies showing the importance of Zn and Cu mobilisation in tissues and phagocytic cells with respect to clearance of *Salmonella* and *Streptococcus pyogenes* infections [32–35].

Bacterial evasion of metal toxicity is facilitated by a number of distinct mechanisms with the major theme being that of metal efflux [36, 37]. For Cu, the P-type ATPase efflux systems, such as CopA from *Escherichia coli* [38], are highly efficient in exporting the metal from the cytoplasm. Cu resistance is also achieved through the action of the CopB outer membrane protein (OMP), and periplasmic multi-copperoxidases, such as CopA of *Pseudomonas syringae* and CueO of *E. coli* [39, 40]. In Gram-negative bacteria, Cu as well as Zn can be exported by the Heavy Metal Efflux (HME) family of transporters, which are a subclass of the tripartite Resistance-Nodulation cell Division (RND) family of efflux pumps. These are large multimembrane-spanning protein complexes, comprised of inner membrane proteins, periplasmic membrane fusion proteins and OMPs [41, 42]. Together these complexes allow for the export of metal ions across the outer membrane and into the extracellular milieu, thereby preventing intracellular toxicity. Both Zn and Cu are known substrates of the HME efflux systems, with CusCFBA from *E. coli* being a well-characterised example of a Cu-exporting HME transporter [43]. Efflux of metal ions is also achieved via the cation diffusion facilitator (CDF) family of transporters, which includes members capable of exporting Zn and/or Cu. The most well characterised CDF is YiiP from *E. coli*, with a high-resolution crystal structure of the protein providing significant insight into the metal ion translocation pathway of these transporters [44]. Finally, although predominantly known for their role in magnesium transport, various members of the CorA family have also been shown to export Zn, with these proteins classified as ZntB-like proteins [45, 46]. Despite these systems being well characterised in a range of bacterial species, the mechanisms by which *A. baumannii* responds to metal toxicity, particularly that mediated by zinc, have not been elucidated.

Here, we investigated the individual and combined impact of Zn and Cu stress on *A. baumannii*. By combining phenotypic studies with extensive genome and transcriptional analyses, we identified genes that play a role in resistance to Zn and Cu intoxication and show how metal stress impacts *A. baumannii* physiology.

## Methods

### Bacterial strains, chemicals, media and growth

**Fig. 1:**
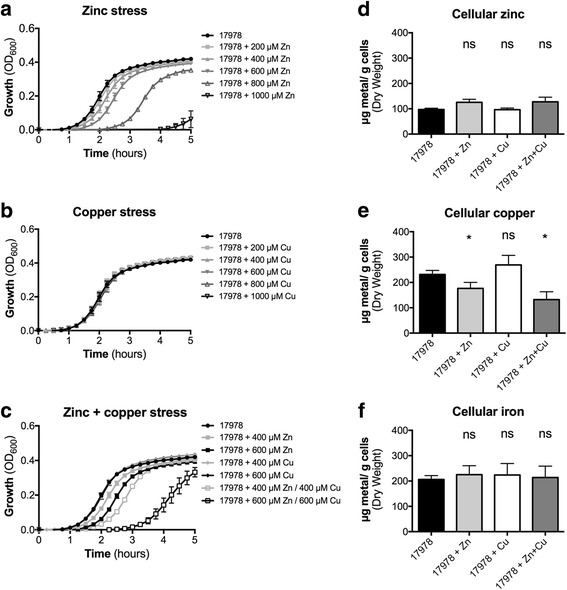
The effect of Zn and Cu stress on *A. baumannii* growth and metal ion homeostasis. Growth as determined by measuring the optical density at 600 nm (OD_600_) of *A. baumannii* strain ATCC 17978 under increasing concentrations of (**a**) Zn, (**b**) Cu, or (**c**) Zn + Cu (*n* ≥ 3). Examination of the (**d**) Zn, (**e**) Cu and (**f**) Fe levels in untreated cells and cells grown in the presence of 400 μM Zn, 400 μM Cu or 400 μM Zn + 400 μM Cu. The cellular metal ion content (in microgram) per gram of desiccated cells was determined by ICP-MS. The data are the mean of at least biological triplicates (± SEM). Statistical analyses were performed using a two-tailed Student’s *t*-test; ns = not significant and * = *p* < 0.05

The *Acinetobacter* strains used in this study have been described previously [4]. All chemicals were purchased from Sigma Aldrich unless otherwise indicated. *A. baumannii* strains were routinely grown in Luria Bertani broth (LB), containing 1% tryptone (BD Bacto), 0.5% yeast extract (BD Bacto) and 1% sodium chloride. For routine overnight culturing of *A. baumannii* strains, a single colony from LB agar was used to inoculate 5 mL of LB medium. Overnight cultures were diluted to an optical density at 600 nm (OD_600_) of 0.01 in either 200 μL for growth assays or 20 mL for all other analyses. For growth assays, cultures in LB media were incubated at 37 °C with shaking in a FLUOStar Omega Spectrophotometer (BMG Labtech), with the OD_600_ values presented (Fig. 1 and Additional file 1). To assess the impact of ZnSO_4_ and/or CuSO_4_ stress, paraquat, hydrogen peroxide or arachidonic acid on *A. baumannii* fitness, EC_50_ values were calculated from the OD_600_ measurements using Graphpad Prism 6.0, and then compared between treated and untreated cultures (Figs. 4, 5a, d and 6e). The 20 mL cultures used for all other analyses were incubated at 37 °C in an Innova 40R shaking incubator (Eppendorf) at 230 rpm until they reached mid log-phase (OD_600_ = 0.7).

### Cellular metal ion content analysis

Untreated and metal-stressed bacteria (LB supplemented with 400 μM ZnSO_4_, 400 μM CuSO_4_, or 400 μM ZnSO_4_ and 400 μM CuSO_4_) were harvested at mid log-phase and washed, by resuspension and centrifugation at 7000 × *g* for 8 mins, three times with phosphate-buffered saline (PBS) containing 5 mM ethylenediaminetetraacetic acid (EDTA), and then 6 times with PBS as described previously [47–50]. Bacterial pellets were desiccated at 95 °C overnight. The dry cell weight was measured and the pellets resuspended in 35% HNO_3_ and boiled at 95 °C for 1 h prior to removal of debris by centrifugation. Samples were diluted to a final concentration of 3.5% HNO_3_ and analysed by inductively coupled plasma-mass spectrometry (ICP-MS) on an Agilent 7500cx ICP-MS (Adelaide Microscopy, University of Adelaide). Results are the mean (± SEM) of at least three independent experiments, with the statistical significance determined using a two-tailed Student’s *t*-test.

### Comparative genomics

Comparative BLASTP (2.2.28+) searches [51] of all annotated protein coding sequences in *A. baumannii* strains ATCC 17978, ATCC 19606T, D1279779, SDF, ACICU, AB0057, WM99c and 6870155, and *Acinetobacter baylyi* ADP1 were executed through the Proteinortho tool [52] to identify putative orthologous/paralogous proteins in each of the strains. Transport protein predictions were made using the Transporter Automated Annotation Pipeline (TransAAP [53]) to identify putative Zn and Cu transporters. Where orthologous/paralogous efflux transporters were identified between/within strains, genetic maps demonstrating the conservation of their coding gene and surrounding genes were generated using the Easyfig tool [54].

### RNA isolation and qRT-PCR

For RNA extraction and qRT-PCR analysis, untreated or metal-stressed bacteria (LB supplemented with 400 μM ZnSO_4_, 400 μM CuSO_4_, or 400 μM ZnSO_4_ + 400 μM CuSO_4_) were harvested at mid log-phase and lysed in QiaZol (Qiagen) as described previously [55, 56]. Following the addition of chloroform and phase separation, RNA was extracted and purified using a PureLink RNA Mini Kit (Thermo Fisher Scientific), according to the manufacturer’s instructions. The total RNA samples were treated with DNase I (Roche) and qRT-PCR performed using the SuperScript III One-Step RT-PCR kit (Thermo Fisher Scientific) on a LC480 Real-Time Cycler (Roche). Transcription levels of genes were corrected to those obtained for *GAPDH* prior to normalization to the transcription levels observed for untreated *A. baumannii* cultures. Primer sequences are listed in Additional file 2. Results are the mean (± SEM) of at least three independent experiments, with the statistical significance determined using a one-way ANOVA with Dunnett’s post-test.

### Superoxide dismutase activity assay

Untreated and metal-stressed bacteria (LB supplemented with 400 μM ZnSO_4_, 400 μM CuSO_4_, or 400 μM ZnSO_4_ + 400 μM CuSO_4_) were harvested at mid log-phase (OD_600_ = 0.7), washed in PBS and lysed by sonication on a BioRuptor (Diagenode). Following removal of cell debris by centrifugation, the SOD activity was determined according the manufacturer’s protocol (Cayman). The data was corrected for the total protein input followed by normalisation against levels determined for untreated cells. The data are the means of at least biological triplicates (± SEM), with the statistical significance determined using a Student’s *t*-test.

### Fatty acid analyses

Untreated or metal-stressed bacteria (LB supplemented with 400 μM ZnSO_4_, 400 μM CuSO_4_, or 400 μM ZnSO_4_ + 400 μM CuSO_4_) were harvested at mid log-phase and analysed by gas chromatography at the School of Agriculture, Food and Wine, University of Adelaide, as previously described [9, 57]. The data are the mean of five biological replicates (± SEM), with the statistical significance determined using a one-way ANOVA with Dunnett’s post-test.

### Macrophage killing assays

THP-1 cells (ATCC TIB-202) were grown under atmospheric control (5% CO_2_) at 37 °C in complete RPMI medium (RPMI with phenol red [Thermo Fisher Scientific], supplemented with 10% fetal bovine serum, 10 mM HEPES, 30 μg.mL^−1^ penicillin and 50 μg.mL^−1^ streptomycin). Cell culture flasks (25 cm^2^; BD Falcon) were seeded with 3.5 × 10^6^ THP-1 cells and differentiated by adding 100 ng.mL^−1^ phorbol 12-myristate 13-acetate (PMA), prior to incubation for 3 days. Attached, differentiated, THP-1 cells (macrophages) were washed in complete RPMI and incubated with complete RPMI without added PMA to allow resting for a minimum of 2 days. Prior to challenge with *A. baumannii*, macrophages were detached using 1 mL StemPro Accutase (Thermo Fisher Scientific), washed in Hank’s Balanced Salt Solution (HBSS; Thermo Fisher Scientific) and treated with either 50 μM ZnSO_4_, 50 μM CuSO_4_, or 50 μM ZnSO_4_ + 50 μM CuSO_4_ for 1 h. Metal-supplemented macrophages were washed and diluted to 1.1 × 10^5^ cells mL^−1^ in HBSS. *A. baumannii* cultures grown to mid-log phase (OD_600_ = 0.7) were washed in HBSS and co-incubated with the macrophages at a multiplicity of infection of 10 for 60 min. The colony forming units (CFUs) of *A. baumannii* were determined by plating onto LB agar plates and incubation at room temperature for 24 h. The data was normalised for CFUs in the presence of untreated macrophages. The data are the mean of three biological replicates (± SEM). The statistical differences between the CFUs determined in the presence of untreated and metal-treated macrophages were examined using a two-tailed Student’s *t*-test.

## Results

### Zn stress abrogates *A. baumannii* ATCC 17978 Cu homeostasis

We assessed the impact of extracellular Zn and Cu ions on *A. baumannii* fitness by examining growth of *A. baumannii* strain ATCC 17978 in the presence of increasing concentrations of ZnSO_4_ and/or CuSO_4_ (200, 400, 600, 800 and 1000 μM). We observed that *A. baumannii* ATCC 17978 was acutely sensitive to extracellular Zn abundance with 400 μM Zn supplementation resulting in a ~30 min growth delay (Fig. 1a). Bacterial fitness decreased further at higher Zn concentrations, with growth delays of approximately 1 and 2.5 h observed in 600 and 800 μM Zn, respectively. By contrast, Cu stress had no apparent phenotypic effect on *A. baumannii* ATCC 17978 at any of the concentrations examined (Fig. 1b). The limited impact of 1000 μM Cu on the growth of *A. baumannii* corroborates observations from a previous study which examined copper stress in a nutrient limited media [58]. Treatment with both Zn + Cu had a synergistic impact on *A. baumannii* ATCC 17978 growth (Fig. 1c) as the magnitude of growth delay was greater than that observed for the individual treatments with Zn (Fig. 1a) or Cu (Fig. 1b). This is in contrast to the effect of solid brass (Zn + Cu) on *A. baumannii* survival, which was shown to have a more moderate antimicrobial effect as compared to Cu alone [58].

We then investigated the effects of Zn, Cu and Zn + Cu on metal ion accumulation in *A. baumannii* ATCC 17978 by ICP-MS. Metal content analysis was performed on cells grown in the presence of 400 μM Zn, 400 μM Cu, or 400 μM Zn + 400 μM Cu. The first row transition metal ions Zn, Cu and Fe were the most abundant in *A. baumannii* (Additional file 3; Fig. 1d, e and f). Minor, but not significant (*p* > 0.05), increases in cellular accumulation of Zn were observed when cells were grown in the presence of Zn and Zn + Cu (Fig. 1d). Similarly, treatment with Cu did not result in increased Cu accumulation (Fig. 1e). However, cellular Cu levels were significantly decreased in cells grown in the presence of Zn by comparison to untreated cells (Fig. 1e). Intriguingly, supplementing this condition with Cu, i.e. Zn + Cu treatment, did not rescue the Zn-induced Cu depletion. Instead, the combined treatment resulted in a further decrease in cellular Cu levels, greater than that observed for Zn treatment alone. The impact on Cu accumulation was specific, as the other first row transition metal ions, Mn, cobalt (Co), nickel (Ni) and Fe, were not significantly affected by any of the treatments (Fig. 1e; Additional file 3).

### *A. baumannii* ATCC 17978 possesses a broad arsenal of putative Zn and Cu efflux systems

**Fig. 2:**
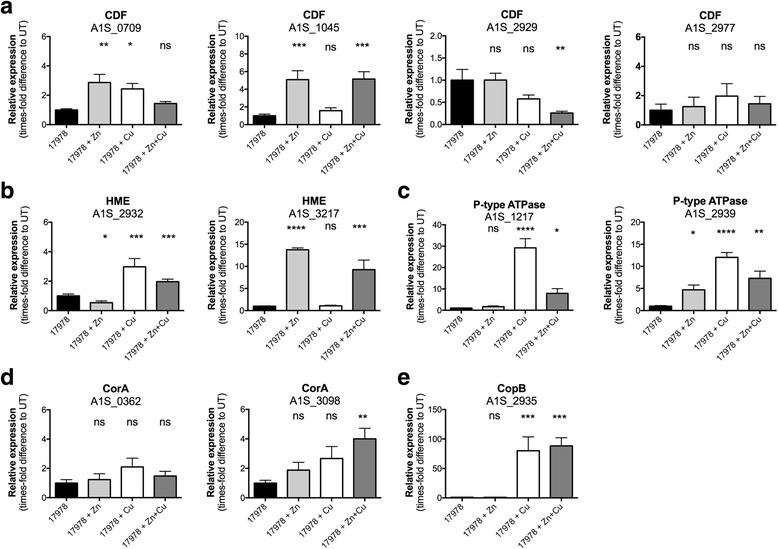
Transcriptional analyses of putative *A. baumannii* Zn and Cu efflux systems. The mRNA transcription levels of the putative (**a**) Cation Diffusion Facilitator (CDF), (**b**) Heavy Metal Efflux (HME), (**c**) P-type ATPase, (**d**) CorA-type and (**e**) CopB-like copper resistance genes of *A. baumannii* strain ATCC 17978 were determined by qRT-PCR. Transcription levels were examined in the presence of 400 μM Zn, 400 μM Cu or 400 μM Zn + 400 μM Cu and corrected to untreated cells following internal normalisation to *GAPDH*. The data are the mean of at least biological triplicates (± SEM). Statistical analyses were performed by one-way ANOVA using Dunnett’s posttest; ns = not significant, * = *p* < 0.05, ** = *p* < 0.01, *** = *p* < 0.001 and **** = *p* < 0.0001

Transporters associated with metal ion efflux serve crucial roles in preventing metal ion intoxication. We observed that in the presence of extracellular Zn and/or Cu stress, *A. baumannii* ATCC 17978 did not accumulate these metals at higher concentrations than untreated cells, which can be attributed, at least in part, to the action of metal ion efflux pathways. Here, we used TransAAP to identify putative Zn and Cu efflux systems encoded in *A. baumannii* ATCC 17978. We identified 13 efflux systems that belong to either the CDF family (TCDB: 2.A.4), P-type ATPase family (TCDB: 3.A.3), CorA metal ion transporter family (TCDB: 1.A.35), HME family of RND transporters (TCDB: 2.A.6.1), or CopB-type family of Cu exporters. These were then examined for their transcriptional responsiveness to Zn and/or Cu stress (400 μM of each ion) as a hallmark of their potential contribution to metal ion homeostasis. We observed that in *A. baumannii* ATCC 17978, extracellular Zn stress induced transcription of the genes encoding four of the 13 putative efflux systems (Fig. 2). The genes up-regulated included two CDF transporters, (A1S_0709, 2.9-fold; and A1S_1045, 5.0-fold), one HME efflux system (A1S_3217, 13.7-fold) and one P-type ATPase (A1S_2939, 4.7-fold). Growth in the presence of Cu stress resulted in the up-regulation of five of the 13 putative Zn or Cu transport systems. These included the Zn-induced CDF family transporter A1S_0709 (2.4-fold), and the P-type ATPase, A1S_2939 (12.0-fold). In addition, the HME family transporter A1S_2932 (2.9-fold), the CopB-like outer membrane protein A1S_2935 (80.0-fold) and the P-type ATPase A1S_1217 (29.2-fold) were specifically up-regulated in the presence of Cu. Growth in the presence of both Zn and Cu stress induced most, but not all, of the efflux systems observed to be up-regulated under the individual stresses of Zn or Cu (Fig. 2). Intriguingly, although the CDF family transporter A1S_0709, was up-regulated in the presence of the individual ion stress, it was transcriptionally unresponsive in the presence of Zn + Cu. In addition, the CDF family transporter A1S_2929 was significantly down-regulated when cells were stressed with Zn + Cu (3.9-fold), yet transcription was not altered when treated with Zn or Cu alone. By contrast, the CorA-type exporter A1S_3098 that was unresponsive to the individual Zn or Cu stresses was observed to be up-regulated (4.0-fold) when treated with both ions.

Our observations corroborate “omics” analyses of Zn and Cu stress in other bacteria, where proteomic/transcriptomic profiles show significant overlap between the two independent stresses [59–61]. Collectively, our data show that *A. baumannii* ATCC 17978 possesses a number of efflux systems associated with resistance to Zn and/or Cu ions. The overlap between Zn and Cu resistance profiles revealed two metal ion efflux systems that may have poly-specific cation efflux properties. However, it is also possible that one or both of these pathways may be Cu-specific and activated inappropriately under Zn stress, resulting in the Cu-depletion phenotype observed.

### Comparative analysis of Zn and Cu resistance mechanisms in diverse *Acinetobacter* strains

**Fig. 3:**
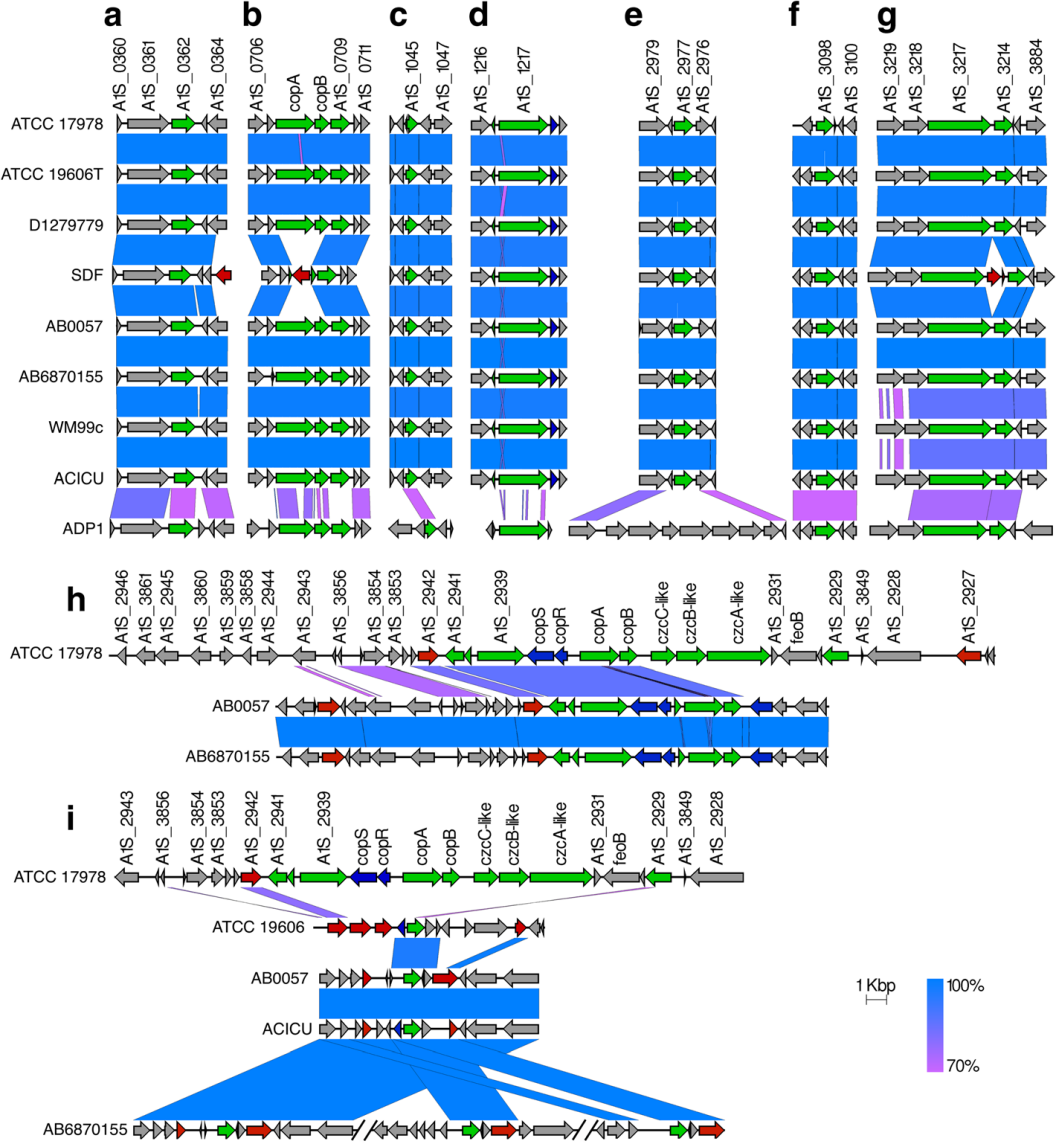
Comparative analysis of the *Acinetobacter* Zn and Cu efflux systems. Genomic regions containing putative efflux systems of the *Acinetobacter* strains are shown in panels (**a**-**i**). Putative efflux systems were identified using the TransAAP [53] and subsequently aligned and visualised using the Easyfig tool [54]. Genes encoding putative Zn or Cu efflux systems are coloured in green, putative regulators in proximity to these transporters are coloured in blue, genes that may encode for DNA mobilization proteins are coloured red, and other genes are coloured grey. The percentage identity between the regions being compared is indicated by shading

Previous studies of the *A. baumannii* antibiotic resistance and virulence mechanisms have revealed major genomic variation between strains [4, 62]. Therefore, we examined the presence of putative Zn and/or Cu efflux systems across a collection of highly diverse strains previously analysed by our groups [4], as well as the environmental isolate *Acinetobacter baylyi* ADP1. The number of genes encoding putative Zn and/or Cu efflux systems identified ranged between eight (strain SDF) and 18 (strain AB6870155) in each of the strains examined, but all were chromosomally encoded. To determine if the putative efflux systems identified were conserved between strains, and whether conserved transporters were encoded in the same genomic locations, comparative blast searches were conducted between the genomes of the respective strains. These searches revealed a complement of seven genes encoding putative Zn and/or Cu efflux systems that were conserved in all nine of the strains studied, including *A. baylyi* ADP1, albeit at lower sequence identity. These included the CDF transporters orthologous to A1S_0709, A1S_1045 and A1S_3214, P-ATPase system orthologous to A1S_1217, HME system orthologous to A1S_3217, and CorA family transporters orthologous to A1S_0362 and A1S_3098 (Fig. 3). *A. baylyi* ADP1 only encoded one putative Zn and/or Cu efflux system that was not found in the *A. baumannii* strains, a CDF family transporter, ACIAD0449. The significant conservation of Zn and/or Cu efflux proteins between *A. baylyi* ADP1 and the *A. baumannii* strains is notable, and suggests that Zn and Cu efflux are core functions that have been conserved since before the divergence of these species.

*A. baumannii* SDF features the least number of genes encoding putative Zn and/or Cu efflux systems (8), which is likely due to the increased prevalence of insertion sequence elements (IS) and extensive genome degradation in this strain. At least two metal resistance mechanisms found in all other strains (*copA* and *copB*), orthologous to A1S_0707 and A1S_0708 in ATCC 17978, have been deleted or insertionally disrupted in the genome of SDF by an IS (Fig. 3b). These genes appear to be encoded in a single operon with the CDF transporter gene orthologous to A1S_0709. Therefore, expression of this CDF transporter could also be affected by this IS insertion. Additionally, an IS is present in the SDF genome between the genes encoding two conserved putative metal ion efflux systems listed above, orthologs of A1S_3214 and A1S_3217 (Fig. 3g). As a consequence, the expression levels of the A1S_3214 ortholog in strain SDF may be affected by the insertion of this IS into its putative regulatory region. Five strains harboured more than ten genes encoding putative Zn and/or Cu efflux components, including ATCC 17978 (17), ATCC 19606T (11), AB0057 (16), AB6870155 (18), and ACICU (11). Each of these strains carried genes encoding putative Zn and/or Cu efflux systems in chromosomal regions that are likely to have been acquired horizontally on mobile genetic elements. The largest of these elements is a Cu-resistance island (Fig. 3h), which has been previously reported in *A. baumannii* strain LAC-4 [63]. In addition to strain ATCC 17978, strains AB0057 and AB6870155 also encode part of this island, which has inserted into the *dusA* locus [64]. Furthermore, genes orthologous to the CDF family system encoded by A1S_2929 in the ATCC 17978 Cu-resistance island are also found in AB0057, AB6870155, ATCC 19606T and ACICU, but at different loci (Fig. 3i). Notably, AB6870155 carries three copies of this transporter. As mentioned above, AB6870155 encodes the greatest number of putative Zn and/or Cu efflux systems of the strains studied, suggesting that this strain may have frequently encountered elevated levels of metal ions during its evolution. All other Zn and/or Cu resistance mechanisms, i.e. those not associated with the Cu-resistance island, may be considered part of the core metal ion resistome.

### Growth characteristics of diverse *Acinetobacter* strains in the presence of Zn and Cu

**Fig. 4:**
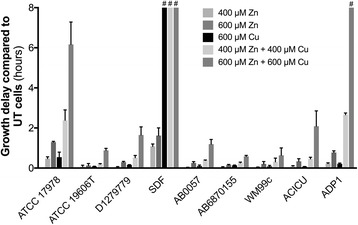
The effects of Zn and/or Cu stress on other *Acinetobacter* strains. Strains of *Acinetobacter* were diluted to OD_600_ = 0.01 from stationary phase cultures and grown without metal ion stress (untreated; UT), or in the presence of 400 μM Zn, 600 μM Zn, 600 μM Cu, 400 μM Zn + 400 μM Cu, or 600 μM Zn + 600 μM Cu. The effect of metal ion stress was examined by comparing the EC_50_, which was calculated from OD_600_ measurements taken every 6 mins. The results are the average of at least four replicate cultures of at least two distinct biological samples. ^#^ indicates no growth detected within 18 h

Given the differences in the number and type of putative metal ion efflux systems encoded by the *Acinetobacter* strains, we investigated whether this had a phenotypic impact on their resistance profiles to Zn and/or Cu. Here, we examined growth of each of the *A. baumannii* strains and *A. baylyi* ADP1 in the presence of 400 μM Zn, 600 μM Zn, 400 μM Cu, 600 μM Cu, 400 μM Zn + 400 μM Cu, and 600 μM Zn + 600 μM Cu. Although growth in the presence of 400 μM Cu was examined, the majority of *A. baumannii* strains were unaffected by this concentration and so we have excluded this condition from further analysis. We observed that all strains experienced a growth delay in the presence of 600 μM Zn (Fig. 4). However, with the exception of the SDF strain, the Zn-induced growth delays were generally less than that observed for the ATCC 17978 strain. This was unexpected and cannot be solely explained by comparative analyses of the Zn efflux systems described above. The other strains also showed resistance to extracellular Cu comparable to the ATCC 17878 strain. However, the SDF strain was a notable exception with no growth observed in the presence of 600 μM Cu (Fig. 4). This suggests that the *copA* and *copB* Cu resistance mechanisms (orthologs of A1S_0707 and A1S_0708, respectively, in the ATCC 17978 strain), which are disrupted by IS elements, could have a prominent role in *Acinetobacter* Cu resistance (Fig. 3). The combination of Zn + Cu stress resulted in a synergistic growth delay in most *Acinetobacter* strains. Although, similar to the impact of Zn stress, the majority of strains were affected less by Zn + Cu stress by comparison with the ATCC 17978 strain. The sole exception was the SDF strain, which was hyper-susceptible to the combination of Zn + Cu stress.

Taken together, these data suggest that CopA/CopB-type copper resistance mechanisms have a prominent role in Cu tolerance in *Acinetobacter* species, based on the reduced Cu resistance in strain SDF. However, the genomic and phenotypic analyses do not reveal a specific efflux system or type of efflux pathway as being a major determinant of Zn resistance. As the combination of metal ion stresses acted in a synergistic manner in all strains, the underlying cause of this phenotype appears to be conserved amongst all strains.

### Zn and Cu contribute to *A. baumannii* ATCC 17978 resistance to reactive oxygen species

Metal ion intoxication is often associated with an increased risk of susceptibility to intracellular reactive oxygen species (ROS). Zinc, although not directly redox active, has been proposed to contribute to oxidative stress via the displacement of Fe from Fe-S cluster-containing proteins [21]. By contrast, Cu, which is reduced to Cu(I) in the cytoplasm, is highly redox active and acutely toxic at elevated intracellular concentrations [65]. Despite this, both Zn and Cu ions are crucial co-factors in the Cu-Zn superoxide dismutase (Cu-Zn-SOD), which detoxifies superoxide anions (O_2_^.-^). Thus we sought to examine the impact of extracellular Zn and/or Cu on the resistance of *A. baumannii* to O_2_^.-^ and H_2_O_2_. Here, we examined the growth phenotype of *A. baumannii* ATCC 17978 in the presence of 400 μM Zn and/or Cu and oxidative stress.

**Fig. 5:**
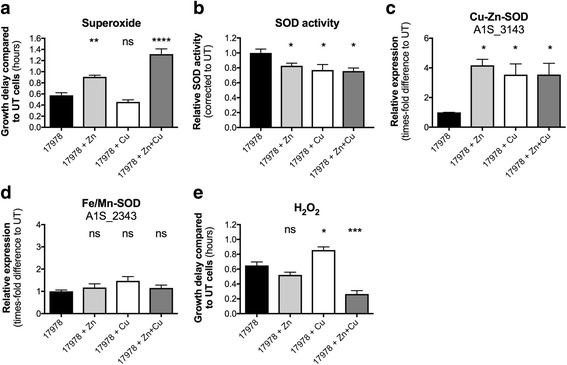
The effect of Zn and Cu treatment on the resistance of *A. baumannii* to oxidative stress. The effect of (**a**) 40 μM paraquat and (**e**) 160 μM H_2_O_2_ on *A. baumannii* strain ATCC 17978 grown in the presence of 400 μM Zn, 400 μM Cu, 400 μM Zn + 400 μM Cu, or without added metal ions, was determined by measuring the optical density at 600 nm (OD_600_). The growth delay between untreated (UT) and paraquat- or H_2_O_2_-treated cells was examined by comparing the EC_50_ under each relevant metal ion stress condition. Statistical analyses were performed using a one-way ANOVA. **b** The total SOD activity of mid-log phase cells (untreated, 400 μM Zn, 400 μM Cu or 400 μM Zn + 400 μM Cu) was determined. Data were corrected for total protein content, followed by normalisation to untreated cells. Statistical analyses were performed using a Student’s *t*-test. The mRNA transcription levels of (**c**) Cu-Zn superoxide dismutase (Cu-Zn-SOD; A1S_3143), (**d**) Fe/Mn superoxide dismutase (Fe/Mn-SOD; A1S_2343) were determined by qRT-PCR. The transcription levels examined in the presence of 400 μM Zn, 400 μM Cu or 400 μM Zn + 400 μM Cu were corrected to untreated cells following internal normalisation to *GAPDH*. The data are the mean of at least biological triplicates (± SEM). Statistical analyses were performed by one-way ANOVA using Dunnett’s posttest. For all statistical analyses; ns = not significant, * = *p* < 0.05, ** = *p* < 0.01, *** = *p* < 0.001 and **** = *p* < 0.0001

Our analyses revealed that Zn-mediated depletion of cellular Cu in *A. baumannii* increased the bacterium’s susceptibility to intracellular O_2_^.-^ (media supplemented with 40 μM paraquat) (Fig. 5a and Additional file 1A). Detoxification of O_2_^.-^ in *A. baumannii* ATCC 17978 is facilitated by a putative Zn-Cu-SOD (A1S_3143) and a previously characterised Fe/Mn SOD (Fe/Mn-SOD) (A1S_2343) [66]. Whilst Cu-Zn-SOD proteins have been reported to localise to the periplasm in other Gram-negative bacteria such as *E. coli* and *Salmonella* [67, 68], the *A. baumannii* Cu-Zn-SOD sequence lacks a signal sequence for periplasmic targeting (Additional file 4), suggesting a cytoplasmic localisation. Interestingly, the total cellular SOD activity was reduced to a similar extent in cells under Zn (17%), Cu (23%) and Zn + Cu stress (24%) (Fig. 5b). Although this does not explain the growth perturbation observed, the total SOD activity inversely correlates with the transcription levels of the Cu-Zn-SOD, which was found to be up-regulated to a similar extent under all metal stress conditions examined (~4-fold; Fig. 5c). Transcriptional analysis of the Fe/Mn-SOD did not reveal any differences (Fig. 5d). Metal stress was also demonstrated to significantly affect the resistance of *A. baumannii* to H_2_O_2_ (Fig. 5e and Additional file 1B). Cu supplementation of *A. baumannii* resulted in a significant increase in sensitivity of H_2_O_2_, with Cu depletion (via Zn or Zn + Cu supplementation) causing a significant decrease in H_2_O_2_ sensitivity. Collectively, our analyses show that both Zn and Cu ions significantly influence oxidative stress management in *A. baumannii* ATCC 17978.

### Zn and Cu play key roles in modulating *A. baumannii* membrane biology

**Fig. 6:**
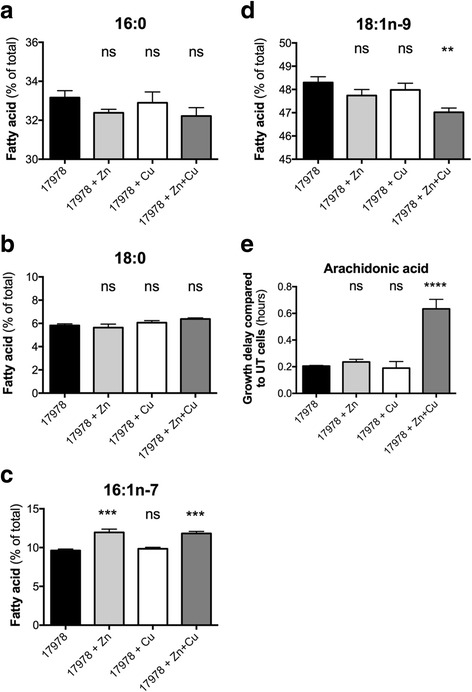
The effects of Zn and Cu on *A. baumannii* membrane biology. The major cellular fatty acid constituents of *A. baumannii* strain ATCC 17978, grown in the presence of 400 μM Zn, 400 μM Cu, 400 μM Zn + 400 μM Cu, or without added metal ions were determined by gas chromatography. The abundance of fatty acids, expressed as percentage of total cellular fatty acids, is (**a**) 16:0 (**b**) 18:0, (**c**) 16:1n-7 and (**d**) 18:1n-7. The data are the mean of at least biological triplicates (± SEM). Statistical analyses were performed by one-way ANOVA using Dunnett’s posttest; ns = not significant, ** = *p* < 0.01 and *** = *p* < 0.001. **e** The susceptibility of *A. baumannii* strain ATCC 17978 to arachidonic acid (20:4n-6) grown in the presence of 400 μM Zn, 400 μM Cu, 400 μM Zn + 400 μM Cu, or without added metal ions was determined by measuring the optical density at 600 nm (OD_600_). The growth delay between untreated and arachidonic acid-treated cells was examined by comparing the EC_50_ under each relevant metal ion stress condition. The data are the mean of at least biological triplicates (± SEM). Statistical analyses were performed by one-way ANOVA using Dunnett’s posttest; ns = not significant and **** *p* < 0.0001

Bacterial cell membranes serve a key role in defence against environmental stresses. Metal ions and oxidative stresses can exert significant and potentially deleterious effects on bacterial cell membrane composition and integrity [69]. Predominantly, this arises through modulation of abundance of unsaturated fatty acids, which impacts membrane fluidity. Here, we sought to examine the impact of metal ion stress on the fatty acid content of mid log-phase *A. baumannii* ATCC 17978 cells grown in presence of 400 μM Zn and/or Cu (Fig. 6a–d). Our analyses showed that *A. baumannii* ATCC 17978 membranes consist of four major fatty acids, which include two saturated fatty acids (16:0 and 18:0) and two mono-unsaturated fatty acids (16:1n-7 and 18:1n-9) (Fig. 6a–d) [9]. Metal ion stress caused significant alterations in the abundance of the mono-unsaturated fatty acids. Zn and Zn + Cu stress induced a significant increase in the abundance of 16:1n-7 mono-unsaturated fatty acids. By contrast, only the combination of Zn + Cu stress was associated with a significant reduction (1.3%) in the abundance of 18:1n-9 mono-unsaturated fatty acids, by comparison with untreated cells. Even these relatively minor changes in the fatty acid composition of the cell membrane will influence membrane fluidity and have been proposed to be one mechanism of bacterial adaption to extracellular stress [69]. Accordingly, we examined how alterations in cell membrane composition, as a function of metal ion stress, influenced resistance to arachidonic acid, a long chain polyunsaturated fatty acid with known antimicrobial activity [70]. Consistent with the observations of metal ion stress on membrane fatty acid composition, *A. baumannii* grown in the presence of 400 μM Zn + Cu showed increased susceptibility to arachidonic acid, with a significantly increased growth delay, by comparison to untreated cells (Fig. 6e and Additional file 1C). Whether this is directly attributable to the decreased abundance of the 18:1n-9 mono-unsaturated fatty acids in these membranes requires further investigation.

### Zn and Cu contribute to macrophage-mediated killing of *A. baumannii*

**Fig. 7:**
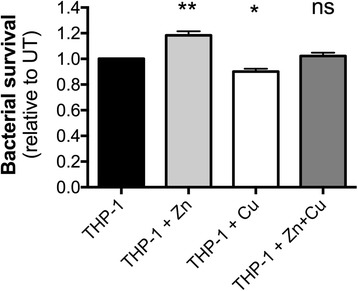
The effect of Zn and Cu on macrophage-mediated killing of *A. baumannii*. The survival of *A. baumannii* ATCC 17978 cells in the presence of THP-1 human monocyte-derived macrophages that were either treated with 50 μM Zn, 50 μM Cu or 50 μM Zn + 50 μM Cu was compared to survival of *A. baumannii* ATCC 17978 cells in the presence of untreated THP-1 cells. The data are the mean of biological triplicates (± SEM). Statistical analyses were performed using a two-tailed Student’s *t*-test; ns = not significant, * = *p* < 0.05 and ** = *p* < 0.01

Macrophages are known to utilize a broad range of antimicrobial compounds to prosecute killing of bacterial pathogens, including, Zn, Cu, H_2_O_2_, O_2_^.-^, and arachidonic acid. Here, we investigated the effect of Zn and/or Cu on the macrophage-mediated killing of *A. baumannii* ATCC 17978. Human THP-1 derived macrophages were supplemented with the metal ions prior to infection with untreated *A. baumannii* cells. We observed that Zn-treated macrophages were significantly compromised in their ability to kill *A. baumannii*, resulting in an 18% higher survival rate, by comparison to bacteria exposed to untreated macrophages (Fig. 7). By contrast, Cu-treated macrophages showed an improvement in bacterial killing (10% lower bacterial survival rate), by comparison with untreated macrophages (Fig. 7). However, macrophages treated with Zn + Cu did not show a significant alteration in the efficiency of bacterial killing. Overall, these data indicate that the Zn and Cu status of macrophages can directly influence the killing of *A. baumannii*, although the molecular basis is likely to be highly complex and warrants significant further investigation.

## Discussion

Bacteria tightly regulate the intracellular concentrations of metal ions to balance their necessity for a range of cellular functions, against the lethal consequences of metal overload and intoxication. In this study, we examined the effects of Zn and Cu toxicity on *A. baumannii* fitness and identified numerous molecular mechanisms associated with management of these stresses, including a range of putative metal ion efflux systems. Exponential growth of *A. baumannii* strain ATCC 17978 was delayed to a greater extent by Zn than by Cu, a phenotype that was shared by six other clinical *A. baumannii* species from different clonal groups, and by the environmental strain *A. baylyi* ADP1. Only strain SDF, isolated from a human associated louse, was found to be more susceptible to Cu than to the equivalent concentration of Zn, which may be attributable to the loss of the *copAB* genes in this strain. Examination of cellular metal ion abundance in strain ATCC 17978 showed that under Zn or Cu stress, neither metal was accumulated at significantly higher levels. This indicates that *A. baumannii* either prevents their uptake or is able to efficiently efflux the ions from the cell.

**Fig. 8:**
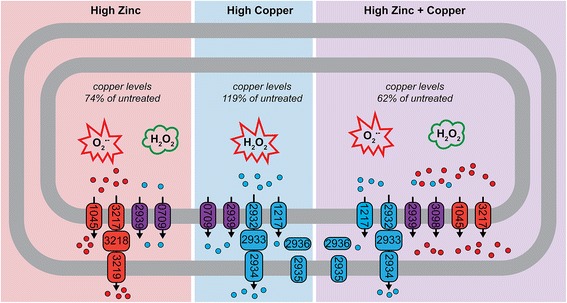
An overview of the cellular responses of *A. baumannii* to Zn and Cu stress. Cartoon representation of *A. baumannii* ATCC 17978 under either high Zn (400 μM), high Cu (400 μM) or high Zn + Cu (400 μM each) conditions based on the observations from this work. The numbers within the efflux systems (ovals) are the locus-tags for *A. baumannii* strain ATCC 17978, excluding their prefix “A1S_”. The efflux proteins in red are those up-regulated under high Zn, in blue by high Cu and those in purple were found to be transcriptionally responsive to both high Zn and Cu independently (A1S_2939 and A1S_0709) or the combination of high Zn and high Cu (A1S_2939 and A1S_3098). Under high Zn and high Zn + Cu conditions, *A. baumannii* becomes increasingly sensitive to superoxide stress (O_2_^.-^ in the red burst), but shows increased resistance to hydrogen peroxide (green cloud). Under high Cu conditions, *A. baumannii* shows increased susceptibility to hydrogen peroxide (H_2_O_2_ in the red burst). Under high Zn conditions and high Zn + Cu conditions, Cu levels reduce to 74 and 62%, respectively

Species of the *Acinetobacter* genus have adapted to survive in a wide variety of environments, which is likely to have contributed to the presence of the substantial number of metal ion efflux systems within their genomes. Using our comprehensive bioinformatic membrane transporter identification pipeline, we identified a large number of these efflux systems, which may be responsible for translocating Zn and/or Cu from the cytoplasm to the periplasm and/or extracellular space in *A. baumannii*. Interestingly, subsequent comparative analyses demonstrated that most of these putative metal resistance systems are highly conserved between diverse *A. baumannii* strains, as well as with the environmental isolate *A. baylyi* ADP1. This suggests that these systems have played a crucial role in survival since the divergence of these *Acinetobacter* species. Nonetheless, some strains encoded considerably more putative metal ion efflux systems than others, typically on putative mobile elements that have been inserted into the chromosome. This additional metal ion efflux capacity suggests that their ancestral strains may have existed in metal-contaminated environments. Insight into the likely substrate specificities of the putative metal ion efflux systems was gained by transcriptional profiling in response to Zn and/or Cu stress. Based on transcriptional activation, the CDFs A1S_0709 (~3-fold up-regulated) and A1S_1045 (~5-fold up-regulated), the P-type ATPase A1S_2939 (~4-fold up-regulated) and the HME A1S_3217 (~14-fold up-regulated) are likely to be involved in Zn efflux in *A. baumannii* strain ATCC 17978 (Figs. 2 and 8). However, since A1S_0709 and A1S_2939 are each positioned in the same loci as Cu-resistance mechanisms and both genes are also transcriptionally activated by Cu stress (~2-fold and ~12-fold, respectively), their importance in Zn export is questionable. Rather, based on transcriptional profiling and conservation across the species, and to some extent genus, our analyses suggest the major Zn efflux systems in *A. baumannii* to be the CDF encoded by A1S_1045 and the HME family system encoded by A1S_3217.

Strain ATCC 17978 possesses three distinct putative Cu-resistance mechanisms on its proposed Cu-resistance island, including the P-type ATPase A1S_2939 (~12-fold up-regulated), HME A1S_2932 (~3-fold upregulated) and CopB A1S_2935 (~80-fold up-regulated) (Figs. 2 and 8). Although highly transcriptionally responsive to Cu, the Cu-resistance island is unlikely to be essential for survival under high levels of Cu stress, as strain D1279779, which lacks all components of this island, displays a similar Cu resistance profile to the other strains examined in this study. The P-type ATPase A1S_1217 and its putative divergently transcribed Cu chaperone *copZ* (A1S_3627) were heavily up-regulated in response to Cu stress (A1S_1217 30-fold, and A1S_3627 19-fold [data not shown]) and are highly conserved across the strains examined in this study (Fig. 3d). Although this system can also be found in the highly Cu-susceptible strain SDF, a potentially critical difference between the SDF strain and all other strains included in this study, is the lack of a CopAB Cu-resistance mechanism (either A1S_0707–A1S_0708 or A1S_2936–A1S_2935). Therefore, the periplasmic Cu oxidase/cupredoxin (CopA) and the outer membrane exporter (CopB) appear critical for dealing with the Cu translocated from the cytoplasm to the periplasm by the inner membrane P-type ATPase A1S_1217.

Previous studies have shown that growth perturbations induced by metal ion stress can be attributed to disruption of cellular metal ion homeostasis, commonly resulting from protein mismetallation [27, 71]. Upon examination of the effect of Zn or Cu stress on the abundance of other first row transition metals, we found that Zn caused a significant depletion of Cu in *A. baumannii*, whereas high extracellular Cu did not significantly affect cellular metal ion levels. The effect of Zn stress on cellular Cu was found to be specific, as the accumulation of other metal ions, such as Fe and Mn, was not altered. Zn toxicity is a widely studied topic, but to our knowledge this is the first report of Zn causing an imbalance in cellular Cu levels in any organism.

To determine whether Cu depletion in the presence of elevated Zn abundance could be rescued by the addition of excess Cu, we examined the effect of equimolar concentrations of Zn and Cu (400 μM) on *A. baumannii* growth and metal ion homeostasis. Surprisingly, instead of an increase in Cu, cellular Cu levels in *A. baumannii* strain ATCC 17978 were further decreased by comparison to cells treated with Zn alone. These findings provide a potential explanation for the observed growth perturbations, which indicated that Zn and Cu have synergistic antimicrobial properties against *A. baumannii*, a phenotype highly conserved across the species and genus. The synergistic toxicity of Zn and Cu has been studied in many different organisms, since these two metals are often both found at elevated concentrations in industrial waste as well in agricultural pesticides [23, 72, 73].

The molecular basis for the Cu depletion observed in Zn-stressed cells could be due to a failure of the cells to acquire adequate amounts of Cu. To date, specific Cu uptake systems have not been identified in the cytoplasmic membrane of *A. baumannii*. Cytoplasmic Cu accumulation may occur via polyspecific uptake system(s) that also recognise other cations such as Zn. Therefore, one possible explanation for the observed impact of Zn stress on Cu accumulation could be due to Zn competing for Cu uptake and/or decreased transcription of these uptake systems in the presence of elevated Zn. However, while this model could plausibly explain the phenotype observed in the presence of high Zn, it is inconsistent with the impact on Cu accumulation that occurred when *A. baumannii* was exposed to equimolar concentrations of Zn and Cu. Based on our transcriptional analyses, we propose that the molecular basis for Zn-induced Cu depletion in *A. baumannii* is due to the up-regulation of efflux systems that preferentially efflux Cu, a phenomenon similar to that seen for the effect of cadmium stress on Zn levels in *S. pneumoniae* [71]. Here, by combining phenotypic data and genomic observations from multiple *A. baumannii* species we can propose roles and contributions of various transporters to the metal resistome of this organism.

Interestingly, three resistance mechanisms were found to respond specifically to Cu, but not Zn (A1S_1217, A1S_2932 and A1S_2935). These genes were also up-regulated in *A. baumannii* under Zn + Cu stress, despite the significant Cu depletion seen in these cells (Figs. 2 and 8). The genes encoding the periplasmic CopAB-type Cu-resistance mechanism (A1S_2936 – A1S_2935) are divergently transcribed from a two-component regulatory system (A1S_2937 – A1S_2938), which typically sense their activating ligands in the periplasmic space. Indeed, A1S_2935 (*copB*) was the only Cu-resistance gene expressed at the same level in Zn + Cu stressed cells as that seen in Cu-stressed cells only, which could indicate that Cu is accumulated at similar levels in the periplasm under Cu and Zn + Cu stress. Consistent with these observation, studies from *E. coli* have shown that periplasmic Cu levels affect transcription of transporters responsible for exporting Cu from the cytosol to the periplasm in a complex regulatory hierarchy that includes both cytosolic (CueR and CopR) and periplasmic (CpxAR, CusRS, and YedWV) Cu-sensing regulators [74–76].

Notably, despite observing a growth delay in the Zn and Zn + Cu stressed cultures at mid-log-phase, their logarithmic growth rate remains unaffected (Fig. 1). This indicates that once the cells have adjusted to a high Zn environment during the lag-phase, their subsequent overall fitness is similar to that of untreated cells. In line with these findings, cells pre-treated with Zn overnight no longer exhibit a growth delay upon subsequent Zn stress (data not shown), similar to that observed by Williams et al. in the case of Cu stress [58]. Thus, Cu depletion does not appear to have major effects on the *A. baumannii* growth rate and may simply be a Zn stress adaptation strategy instead of an undesired consequence.

As seen in many other bacteria, metal ions play a key role in *A. baumannii* resistance to reactive oxygen species. Our examination of *A. baumannii* tolerance to H_2_O_2_ showed that cellular Cu levels play a key role in susceptibility to H_2_O_2_ toxicity. Conversely, high Zn levels are detrimental when *A. baumannii* encounters superoxide. Although not previously characterized, the *A. baumannii* genome features a gene encoding Cu-Zn-SOD. In homologous enzymes, the Cu co-factor is essential for reaction efficiency, with Zn fulfilling structural roles. Hence, under Zn stress, i.e. Cu limitation, this enzyme may fail to aid in the detoxification of superoxide, resulting in oxidative stress. However, due to the similar effect of Cu stress on SOD activity and expression of the Cu-Zn-SOD, the relative roles of the two *A. baumannii* SODs under metal stress require further examination. The significance of Cu and Zn as enzymatic co-factors was also suggested through examination of the bacterial membrane under Zn + Cu stress. Production of the 18:1n-9 fatty acid requires a Cu/Fe-dependent delta-9 desaturase enzyme. This may explain the decreased abundance of this fatty acid in the *A. baumannii* membrane under Cu-depleted conditions and the reduced tolerance to arachidonic acid.

Within macrophages, Zn, Cu, H_2_O_2_, O_2_^.-^ and arachidonic acid have all been shown to be involved in killing pathogens, but whether a combination of these agents are employed simultaneously remains largely unknown and may be bacterium specific. Our analyses revealed that Cu enhances the ability of macrophages to kill *A. baumannii* and that Zn inhibits this action, thereby fitting the hypothesis that H_2_O_2_ plays an important role in macrophage-mediated killing of the pathogen. Interestingly, macrophages supplemented with Zn + Cu displayed killing activity similar to that seen for untreated cells. This may be a result of Zn and Cu not being redirected to the same location as the phagocytosed bacteria within the macrophage, i.e. the results represent the average of Zn- or Cu-treated macrophages, similar to that seen for *Salmonella* [32]. Alternatively, the combined effects of improved H_2_O_2_ tolerance, increased arachidonic acid sensitivity, and the general growth delay under Zn + Cu stress may, overall, not reveal any significant differences.

## Conclusions

Collectively, this work reveals the resistance strategies utilised by *A. baumannii* to survive metal ion stress, allowing it to thrive in diverse environments. However, our work also reveals a potential Achilles’ heel, in which cellular Cu levels can be depleted by Zn stress and that this phenotype can be exacerbated by further supplementation with Cu. This weakness could be exploited in the development of novel antimicrobial strategies to target *A. baumannii* in efforts to reduce the disease burden of this pathogen.

## Additional files

Additional file 1: Graphs of the effect of stress on *A. baumannii* growth. Growth as determined by measuring the optical density at 600 nm (OD_600_) of *A. baumannii* strain ATCC 17978 (400 μM Zn, 400 μM Cu or 400 μM Zn + 400 μM Cu) under stress induced by (A) 40 μM paraquat (O_2_^.-^), (B) 160 μM hydrogen peroxide (H_2_O_2_), or (C) 128 μM arachidonic acid (*n* ≥ 3). (TIFF 470 kb)
Additional file 2: Table of oligonucleotides used in this study. (DOCX 75 kb)
Additional file 3: Table of the quantitative analysis of the *A. baumannii* transition metals under zinc and/or copper stress. (DOCX 42 kb)
Additional file 4: Analysis of Zn-Cu-SOD protein sequence. Identification of the *Salmonella enterica* and *Escherichia coli* Zn-CuSOD sequences by SignalP shows that the *A. baumannii* Zn-Cu-SOD lacks the signal sequence required for translocation to the cell’s periplasm. (TIF 6360 kb)
