# Supplementary figures and images for: Zinc stress induces copper depletion in Acinetobacter baumannii
Source: BMC Microbiol. 2017 Mar 11;17:59. doi: 10.1186/s12866-017-0965-y (PMC5346208; doi:10.1186/s12866-017-0965-y)

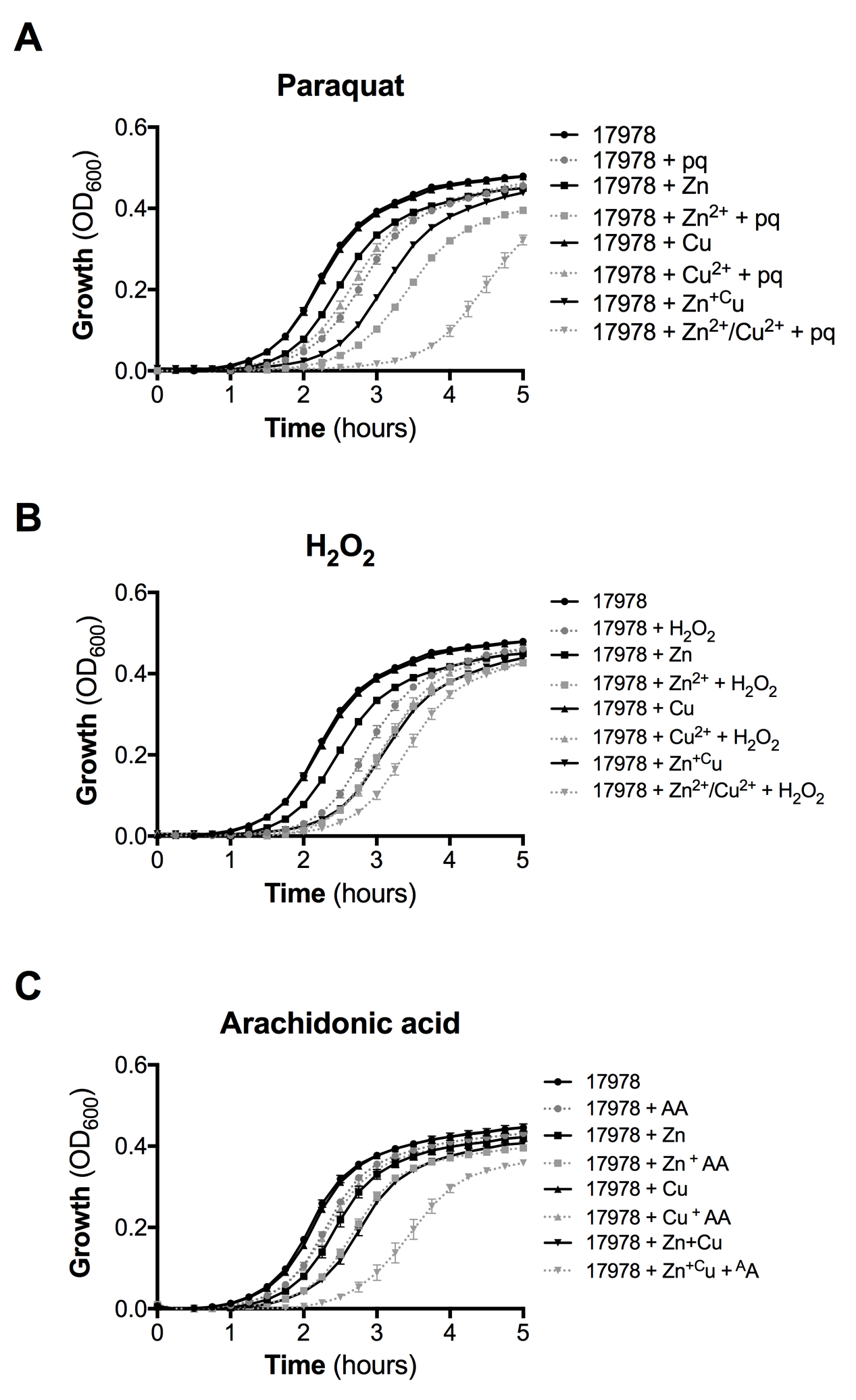

Supplement: Additional file 1: — Graphs of the effect of stress on A. baumannii growth. Growth as determined by measuring the optical density at 600 nm (OD600) of A. baumannii strain ATCC 17978 (400 μM Zn, 400 μM Cu or 400 μM Zn + 400 μM Cu) under stress induced by (A) 40 μM paraquat (O2 .-), (B) 160 μM hydrogen peroxide (H2O2), or (C) 128 μM arachidonic acid (n ≥ 3). (TIFF 470 kb) [file 12866_2017_965_MOESM1_ESM.tiff]

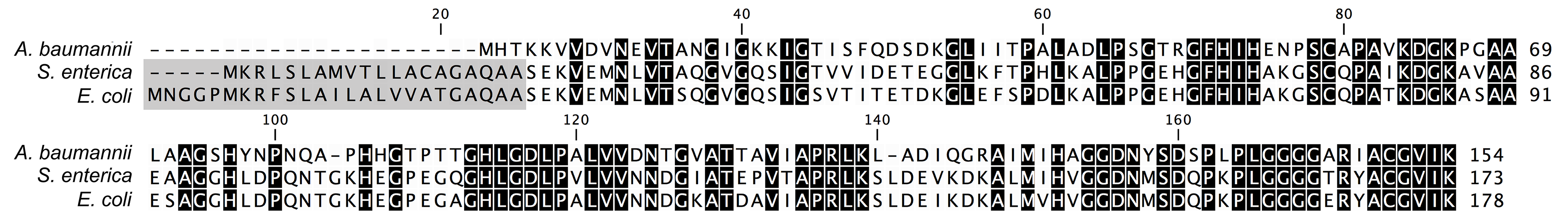

Supplement: Additional file 4: — Analysis of Zn-Cu-SOD protein sequence. Identification of the Salmonella enterica and Escherichia coli Zn-CuSOD sequences by SignalP shows that the A. baumannii Zn-Cu-SOD lacks the signal sequence required for translocation to the cell’s periplasm. (TIF 6360 kb) [file 12866_2017_965_MOESM4_ESM.tif]
