# Supplementary material for: Zinc stress induces copper depletion in Acinetobacter baumannii
Source: BMC Microbiol. 2017 Mar 11;17:59. doi: 10.1186/s12866-017-0965-y (PMC5346208; doi:10.1186/s12866-017-0965-y)
Supplement: Additional file 2: — Table of oligonucleotides used in this study. (DOCX 75 kb) [file 12866_2017_965_MOESM2_ESM.docx]

| **Locus-tag** | **Target** | **Forward (5' -> 3')** | **Reverse (5' -> 3')** |
| --- | --- | --- | --- |
| A1S_0709 | CDF | GGTTCGACCAAGTTCCTCAA | AAATTGCAGCATCGGCAAAC |
| A1S_1045 | CDF | TGCAAAAGTCGTTTGGTCAT | GAATCATTTCGACTACACAA |
| A1S_2929 | CDF | TCGTATGGCATTGTCTCCAG | GCATTTCACCCACAGAGGTG |
| A1S_2977 | CDF | TGACTTCTGTTTTTGCAATT | ACAGGATGGTCCATTTCAGC |
| A1S_3214 | CDF | ATCTCAATGAAAATTCTTAT | TACCGCAATAAGCGTGTCAA |
| A1S_2935 | CopB | CACCTTTTTGGGATTTACAAG | TCAAAACTTGCTCCCCAAAA |
| A1S_0362 | CorA | TTATTCCCGGGCTTAAATCA | CGATCTTGACGATGCTGCTG |
| A1S_3098 | CorA | GAGCTTGCGGATATCTTGGA | AATTAAACAGAGCTGTGCTTT |
| A1S_3143 | Cu-Zn-SOD | TCCACATCCATGAAAATCCA | CCAGTATTGTCAACGACTAAT |
| A1S_2501 | GAPDH | CACCGTCGTACACGTGTTGT | CAACATCACCGCCTTTTTCT |
| A1S_1217 | P-type ATPase | TGGTTGCCGTTGATAAAACA | CTGCTTGAACAATCGCAAGG |
| A1S_2939 | P-type ATPase | AAGAATGGCAGATCAGGTAG | CTAAAGCACACGGACAAGCA |
| A1S_2932 | RND | AAGTGGCTAAACAGGTACCC | TCCAATATTTTCCCCACCAA |
| A1S_3217 | RND | AACTCGCTCCATTTCTCGTT | CCCGTTGAAACAGGAGACAT |
| A1S_2343 | Fe/Mn-SOD | CAATGCTGCTCAAGTTTGGA | TGAGTTGTTGCAGCAGCAGT |
