# Supplementary material for: Zinc stress induces copper depletion in Acinetobacter baumannii
Source: BMC Microbiol. 2017 Mar 11;17:59. doi: 10.1186/s12866-017-0965-y (PMC5346208; doi:10.1186/s12866-017-0965-y)
Supplement: Additional file 3: — Table of the quantitative analysis of the A. baumannii transition metals under zinc and/or copper stress. (DOCX 42 kb) [file 12866_2017_965_MOESM3_ESM.docx]

| **Metal** | **ATCC 17978** | **ATCC 17978 + 400 μM zinc** | **ATCC 17978 + 400 μM copper** | **ATCC 17978 + 400 μM zinc + 400 μM copper** |
| --- | --- | --- | --- | --- |
| Manganese | 2.3 ± 0.4 | 2.42 ± 0.52 | 1.79 ± 0.56 | 1.51 ± 0.43 |
| Iron | 189.5 ± 7.6 | 211.4 ± 16.8 | 203.5 ± 19.3 | 197.5 ± 19.1 |
| Cobalt | 0.12 ± 0.02 | 0.12 ± 0.02 | 0.13 ± 0.03 | 0.09 ± 0.02 |
| Nickel | 3.23 ± 0.39 | 2.39 ± 0.49 | 2.69 ± 0.51 | 1.67 ± 0.29 |
| Copper | 241.2 ± 30.9 | **179.2 ± 19.6** | 290.0 ± 35.8 | **126.0 ± 24.7** |
| Zinc | 97.2 ± 4.9 | 125.5 ± 12.0 | 96.4 ± 6.6 | 127.4 ± 18.5 |
